# Supplementary material for: Subthreshold moment analysis of neuronal populations driven by synchronous synaptic inputs
Source: PLoS Comput Biol. 2025 Oct 31;21(10):e1013645. doi: 10.1371/journal.pcbi.1013645 (PMC12629500; doi:10.1371/journal.pcbi.1013645)
Supplement: S1 Text — (PDF) [file pcbi.1013645.s001.pdf]

Subthreshold moment analysis of neuronal populations driven by synchronous synaptic inputs

Logan A. Becker[1,2], François Baccelli [3,4,5], Thibaud Taillefumier[1,2,3*],

1 Center for Theoretical and Computational Neuroscience, The University of Texas at Austin, Texas, USA
2 Department of Neuroscience, The University of Texas at Austin, Texas, USA
3 Department of Mathematics, The University of Texas at Austin, Texas, USA
4 Departement d'informatique, Ecole Normale Supérieure, Paris, France
5 Institut national de recherche en sciences et technologies du numérique, Paris, France

* ttaillef@austin.utexas.edu

Supporting information

Equation numbering is consistent with the main manuscript. Please refer to the main manuscript for equations (1) to (29).

Appendix 1. Formulas for the shifted moments. Let us consider a neural feedforward population A. For all $a \in A$, we denote the shifted membrane voltage of neuron a by $U_a = V_a - I_a/G_a$. Given a multiset $B = \{a_1, \dots, a_n\}$ of neuron indices in A, our goal here is to compute the shifted stationary moments

$$\mu_B = \mathbb{E}\left[\prod_{a \in B} U_a\right] .$$

To this end, let us adopt the shorthand notations,

$$Q_a = R_a - I_a/G_a \,, \quad R_a = \frac{W_{\mathrm{e},a} V_\mathrm{e} + W_{\mathrm{i},a} V_\mathrm{i}}{W_{\mathrm{e},a} + W_{\mathrm{i},a}} \,, \quad Y_a = e^{-(W_{\mathrm{e},a} + W_{\mathrm{i},a})} .$$

Combining the population-level Marcus update rule Eq. (10) and the time-shift invariance of the Palm distribution Eq. (11), the PASTA principle yields

$$\begin{aligned}
\mu_B &= \mathbb{E}\left[\prod_{a \in B} \left((U_a + J_a) e^{-\frac{S_1}{\tau_a}}\right)\right] , \\
&= \mathbb{E}\left[e^{-\sum_{a \in B} \frac{S_1}{\tau_a}} \prod_{a \in B} \left(U_a + (Q_a - U_a)(1 - Y_a)\right)\right] , \\
&= \mathbb{E}\left[e^{-\sum_{a \in B} \frac{S_1}{\tau_a}}\right] \mathbb{E}\left[\prod_{a \in B} \left(U_a Y_a + Q_a (1 - Y_a)\right)\right] ,
\end{aligned}$$

where the last equation follows from the fact that inter-arrival times are independent from the driven processes. Using the fact that S_1 is exponentially distributed with rate

b, we further obtain by integration the following

$$
\begin{aligned}
\mu_B &= \left(1+\sum_{a\in B}\frac{1}{b\tau_a}\right)^{-1}\mathbb{E}\left[\prod_{a\in B}\left(U_aY_a+Q_a\left(1-Y_a\right)\right)\right], \\
&= \left(1+\sum_{a\in B}\frac{1}{b\tau_a}\right)^{-1}\sum_{C\subset B}\mathbb{E}\left[\left(\prod_{a\in C}U_aY_a\right)\left(\prod_{a\in B\setminus C}Q_a\left(1-Y_a\right)\right)\right], \\
&= \left(1+\sum_{a\in B}\frac{1}{b\tau_a}\right)^{-1}\sum_{C\subset B}\mathbb{E}\left[\prod_{a\in C}U_a\right]\mathbb{E}\left[\prod_{a\in C}Y_a\prod_{a\in B\setminus C}Q_a\left(1-Y_a\right)\right], \qquad (30)
\end{aligned}
$$

where the last equation follows from the fact that the jumps are independent from the driven processes. In order to evaluate the shifted stationary moment μ_B, it is then convenient to introduce the following expectations as coefficients

$$
\gamma_{B;C}=\mathbb{E}\left[\prod_{a\in C}Y_a\left(\prod_{a\in B\setminus C}Q_a\left(1-Y_a\right)\right)\right],
$$

for all multisets $C\subset B$ of indices in A. Then, recognizing the shifted moments in the right-hand side of Eq. (30), the moments μ_B can be determined as the solutions of a triangular system of equations

$$
\mu_B=\left(1+\sum_{a\in B}\frac{1}{b\tau_a}\right)^{-1}\sum_{C\subset B}\gamma_{B;C}\mu_C\,. \qquad (31)
$$

Upon solving for μ_B, the above triangular system leads to the following recursive specification for the moments

$$
\mu_B=\frac{1}{\alpha_B}\left(\sum_{C\subsetneq B}\gamma_{B;C}\mu_C\right),
$$

where the coefficient α_B is given by

$$
\alpha_B=1-\mathbb{E}\left[\prod_{a\in B}Y_a\right]+\sum_{a\in B}\frac{1}{b\tau_a}\,. \qquad (32)
$$

Recursively applying the above formula yields an explicit form for the shifted moments

$$
\mu_B=\sum_{m=1}^{n}\sum_{\varnothing=B_0\subsetneq\ldots\subsetneq B_m=B}\prod_{k=1}^{m}\left(\frac{\gamma_{B_k;B_{k-1}}}{\alpha_{B_k}}\right). \qquad (33)
$$

Appendix 2. First-order moments. Applying Eq. (33) for $A_1=\{a\}$ yields the first-order shifted moments as

$$
\mu_a=\mu_{\{a\}}=\frac{\gamma_{\{a\};\varnothing}}{\alpha_{\{a\}}}=\frac{\mathbb{E}\left[Q_a(1-Y_a)\right]}{1/(b\tau_a)+\mathbb{E}\left[1-Y_a\right]}=\frac{\mathbb{E}\left[\left(R_a-I_a/G_a\right)\left(1-Y_a\right)\right]}{1/(b\tau_a)+\mathbb{E}\left[1-Y_a\right]}.
$$

An alternative and useful expression for μ_a is obtained by observing that

$$
\mathbb{E}\left[Q_a(1-Y_a)\right]=\mathbb{E}\left[(R_a-m_a)(1-Y_a)\right]+\mu_a\mathbb{E}\left[1-Y_a\right].
$$

so that we have

$$\mu_a = b_a \tau_a \mathbb{E}\left[(R_a - m_a)(1 - Y_a)\right] . \tag{34}$$

Remembering that $\mu_a = m_a - I_a/G_a$, we deduce that the mean voltage m_a satisfies

$$m_a = \frac{b\tau_a \mathbb{E}\left[R_a\left(1 - Y_a\right)\right] + I_a/G_a}{1 + b\tau_a \mathbb{E}\left[1 - Y_a\right]} = \frac{c_{\mathrm{e},a} V_{\mathrm{e},a} + c_{\mathrm{i},a} V_{\mathrm{i},a} + I_a/G_a}{1 + c_{\mathrm{e},a} + c_{\mathrm{i},a}} ,$$

where the coefficients $c_{\mathrm{e},a}$ and $c_{\mathrm{i},a}$ are given by

$$\begin{aligned} c_{\mathrm{e},a} &= b\tau_a \mathbb{E}\left[\frac{W_{\mathrm{e},a}}{W_{\mathrm{e},a} + W_{\mathrm{i},a}}\left(1 - e^{-(W_{\mathrm{e},a} + W_{\mathrm{i},a})}\right)\right] , \\ c_{\mathrm{i},a} &= b\tau_a \mathbb{E}\left[\frac{W_{\mathrm{i},a}}{W_{\mathrm{e},a} + W_{\mathrm{i},a}}\left(1 - e^{-(W_{\mathrm{e},a} + W_{\mathrm{i},a})}\right)\right] . \end{aligned} \tag{35}$$

Appendix 3. Generic formulas for the mixed central moments. Our goal here is to derive an characterization of the centered moments

$$M_B = \mathbb{E}\left[\prod_{a \in B} (V_a - m_a)\right] , \tag{36}$$

from the recursive definition of the shifted moment μ_B given by Eq. (33). With the convention that $\prod_{a \in \varnothing} (\cdot)_a = 1$, the shifted moments μ_B can be expressed in terms of the central moments M_B via

$$\mu_B = \mathbb{E}\left[\prod_{a \in B} (V_a - I_a/G_a)\right] = \mathbb{E}\left[\prod_{a \in B} (V_a - m_a + \mu_a)\right] = \sum_{C \subset B} M_C \prod_{a \in B \setminus C} \mu_a ,$$

where we denote the first-order shifted moments $\mu_{\{a\}} = \mu_a$ for simplicity. Injecting the above relation into the recursive definition of the shifted moments

$$\alpha_B \mu_B = \sum_{C \subsetneq B} \gamma_{B;C} \mu_C ,$$

yields a recursive definition for the central moments. Specifically, we have

$$\begin{aligned} \alpha_B \left(M_B + \sum_{C \subsetneq B} M_C \prod_{a \in B \setminus C} \mu_a \right) &= \sum_{C \subsetneq B} \gamma_{B;C} \left(\sum_{D \subset C} M_D \prod_{a \in C \setminus D} \mu_a \right) . \\ &= \sum_{C \subsetneq B} M_C \left(\sum_{C \subset D \subsetneq B} \gamma_{B;D} \prod_{a \in D \setminus C} \mu_a \right) , \end{aligned}$$

so that the moment M_B can be obtained from the lower-order moments M_C, $C \subsetneq B$, via

$$M_B = \frac{1}{\alpha_B} \sum_{C \subsetneq B} M_C \left(\sum_{C \subset D \subsetneq B} \gamma_{B;D} \prod_{a \in D \setminus C} \mu_a - \alpha_B \prod_{a \in B \setminus C} \mu_a \right) , \tag{37}$$

with the convention that $M_\varnothing = 1$. Observe that for all singleton $\{a\}$, we consistently have $M_a = M_{\{a\}} = \mathbb{E}\left[V_a - m_a\right] = 0$. This follows from remembering that $\mu_a = \gamma_{a;\varnothing}/\alpha_a$ by Eq. (33), so that applying (37) for $B = \{a\}$ leads to

$$M_a = M_\varnothing \left(\gamma_{a;\varnothing} - \alpha_a \mu_a\right) = 0 .$$

However, it is hard to interpret (37) for higher order moments. To address this limitation, one can try to simplify (37) by writing the coefficients $\gamma_{B;D}$ featuring in (37) under a centered form:

$$\begin{aligned}\gamma_{B;D} &= \mathbb{E}\left[\prod_{a\in D} Y_a \prod_{a\in B\setminus D} Q_a\left(1-Y_a\right)\right],\\ &= \mathbb{E}\left[\prod_{a\in D} Y_a \prod_{a\in B\setminus D}\left(1-Y_a\right)\prod_{a\in B\setminus D}\left(R_a-m_a+\mu_a\right)\right],\\ &= \sum_{E\subset B\setminus D}\left(\prod_{a\in E}\mu_a\right)\mathbb{E}\left[\prod_{a\in D} Y_a \prod_{a\in B\setminus D}\left(1-Y_a\right)\prod_{a\in (B\setminus D)\setminus E}\left(R_a-m_a\right)\right].\end{aligned}$$

It turns out that injecting the above centered form for $\gamma_{B;D}$ into Eq. (37) leads to a series of simplifications, which we give as intermediary results in Appendix 4. Specifically, setting $F = B\setminus(E\cup D)$, for $C\neq\varnothing$, we show that

$$\begin{aligned}\sum_{C\subset D\subsetneq B}\gamma_{B;D}\prod_{a\in D\setminus C}\mu_a = &-\left(\prod_{a\in B\setminus C}\mu_a\right)\mathbb{E}\left[\prod_{a\in B} Y_a\right]\\ &+\sum_{F\subset B\setminus C}\left(\prod_{a\in B\setminus(C\cup F)}\mu_a\right)\mathbb{E}\left[\prod_{a\in C} Y_a\prod_{a\in F}\left(R_a-m_a\right)\left(1-Y_a\right)\right]\end{aligned} \tag{38}$$

and for $C=\varnothing$, we show that

$$\begin{aligned}\sum_{D\subsetneq B}\gamma_{B;D}\prod_{a\in D}\mu_a &= \left(\prod_{a\in B}\mu_a\right)\mathbb{E}\left[1-\prod_{a\in B} Y_a\right]\\ &\quad+\sum_{\varnothing\subsetneq F\subset B}\left(\prod_{a\in B\setminus F}\mu_a\right)\mathbb{E}\left[\prod_{a\in F}\left(R_a-m_a\right)\left(1-Y_a\right)\right],\\ &= \alpha_B\prod_{a\in B}\mu_a \\ &\quad+\sum_{F\subset B,|F|>1}\left(\prod_{a\in B\setminus F}\mu_a\right)\mathbb{E}\left[\prod_{a\in F}\left(R_a-m_a\right)\left(1-Y_a\right)\right].\end{aligned} \tag{39}$$

where we have used Eq. (34) in the last equation. Using the above expressions will allow us to derive compact expressions for low-order centered moment in Appendix 5.

Appendix 4. Results for intermediary calculations. The goal of this appendix is to simplify the expression of the following quantity

$$\begin{aligned}Q_C &= \sum_{C\subset D\subsetneq B}\gamma_{B;D}\prod_{a\in D\setminus C}\mu_a\\ &= \sum_{C\subset D\subsetneq B}\sum_{E\subset B\setminus D}\left(\prod_{a\in E\cup(D\setminus C)}\mu_a\right)\mathbb{E}\left[\prod_{a\in D} Y_a\prod_{a\in B\setminus D}\left(1-Y_a\right)\prod_{a\in(B\setminus D)\setminus E}\left(R_a-m_a\right)\right].\end{aligned}$$

Set $F=(B\setminus D)\setminus E = B\setminus(E\cup D)$, then F can be any set included in $B\setminus C$ and we have $E = B\setminus(F\cup D) = (B\setminus F)\setminus D$. Introducing F allows one to write the double sum

over the sets D and E as a double sum over the set F and D. Specifically, for all set function f, if $C \neq \varnothing$, we have the following change of set variable

$$\sum_{C \subset D \subsetneq B} \sum_{E \subset B \setminus D} f(D, E) = \sum_{F \subset B \setminus C} \sum_{C \subset D \subset B \setminus F} f(D, (B \setminus F) \setminus D) - f(B, \varnothing) \,. \tag{40}$$

By direct evaluation, the boundary term for our case is found to be:

$$f(B, \varnothing) = \left(\prod_{a \in B \setminus C} \mu_a \right) \mathbb{E} \left[\prod_{a \in B} Y_a \right] .$$

Since $(E \cup D) \setminus C = E \cup (D \setminus C) = B \setminus (C \cup F)$, the remaining sum $S_C = Q_C + f(B, \varnothing)$ can be written as

$$S_C = \sum_{F \subset B \setminus C} \left(\prod_{a \in B \setminus (C \cup F)} \mu_a \right) S_{C,F}$$

where we have defined

$$S_{C,F} = \sum_{C \subset D \subset B \setminus F} \mathbb{E} \left[\prod_{a \in D} Y_a \prod_{a \in B \setminus D} (1 - Y_a) \prod_{a \in F} (R_a - m_a) \right] .$$

The latter sum $S_{C,F}$ can be further evaluated by observing that

$$\begin{aligned} S_{C,F} &= \sum_{C \subset D \subset B \setminus F} \mathbb{E} \left[\prod_{a \in D} Y_a \prod_{a \in B \setminus D} (1 - Y_a) \prod_{a \in F} (R_a - m_a) \right] , \\ &= \mathbb{E} \left[\left(\sum_{C \subset D \subset B \setminus F} \prod_{a \in D} Y_a \prod_{a \in (B \setminus F) \setminus D} (1 - Y_a) \right) \prod_{a \in F} (R_a - m_a)\,(1 - Y_a) \right] , \\ &= \mathbb{E} \left[\prod_{a \in C} Y_a \left(\sum_{H \subset B \setminus (F \cup C)} \prod_{a \in H} Y_a \prod_{a \in (B \setminus (F \cup C)) \setminus H} (1 - Y_a) \right) \prod_{a \in F} (R_a - m_a)\,(1 - Y_a) \right] , \\ &= \mathbb{E} \left[\prod_{a \in C} Y_a \prod_{a \in F} (R_a - m_a)\,(1 - Y_a) \right] , \end{aligned}$$

where the last line follows from the binomial identity. Thus, we find that

$$\begin{aligned} Q_C = &\sum_{F \subset B \setminus C} \left(\prod_{a \in B \setminus (C \cup F)} \mu_a \right) \mathbb{E} \left[\prod_{a \in C} Y_a \prod_{a \in F} (R_a - m_a)\,(1 - Y_a) \right] \\ &- \left(\prod_{a \in B \setminus C} \mu_a \right) \mathbb{E} \left[\prod_{a \in B} Y_a \right] . \end{aligned}$$

The above result can be further simplified for $C = \varnothing$, so that one ultimately obtain:

$$Q_\varnothing = \sum_{\varnothing \subsetneq F \subset B} \left(\prod_{a \in B \setminus F} \mu_a \right) \mathbb{E} \left[\prod_{a \in F} (R_a - m_a)\,(1 - Y_a) \right] + \left(\prod_{a \in B} \mu_a \right) \mathbb{E} \left[1 - \prod_{a \in B} Y_a \right] .$$

These establishes the intermediary results Eq. (38) and Eq. (39) used in Appendix 3.

Appendix 5. Low-order mixed central moments formula. Consider two neurons indexed by a_1, a_2 in A and let us set $B = \{a_1, a_2\}$ in Eq. (37). As by definition of the centered moment, we have $M_a = M_{\{a\}} = 0$ for all a in A, we observe that there is only one nonzero term in Eq. (37) corresponding to $C = \varnothing$, so that

$$M_{a_1,a_2} = \frac{1}{\alpha_{a_1,a_2}} M_\varnothing \left(\sum_{D \subsetneq \{a_1,a_2\}} \gamma_{a_1,a_2;D} \prod_{a \in D} \mu_a - \alpha_{a_1,a_2} \prod_{a \in \{a_1,a_2\}} \mu_a \right) . \quad (41)$$

Using Eq. (39), we have

$$\sum_{D \subsetneq \{a_1,a_2\}} \gamma_{a_1,a_2;D} \prod_{a \in D} \mu_a = \alpha_{a_1,a_2} \prod_{a \in \{a_1,a_2\}} \mu_a + \mathbb{E}\left[\prod_{a \in \{a_1,a_2\}} (R_a - m_a)(1 - Y_a) \right] .$$

Injecting the above relation in Eq. (41) leads to the following compact expression for the centered second-order moments:

$$M_{a_1,a_2} = \frac{\mathbb{E}\left[(R_{a_1} - m_{a_1})(1 - Y_{a_1})(R_{a_2} - m_{a_2})(1 - Y_{a_2})\right]}{1/(b\tau_{a_1}) + 1/(b\tau_{a_2}) + \mathbb{E}\left[1 - Y_{a_1} Y_{a_2}\right]} .$$

For the case of three neurons $B = \{a_1, a_2, a_3\}$, the third-order centered moment can be expressed in terms of four coefficients, corresponding to choosing $C = \varnothing$, $\{a_1, a_2\}$, $\{a_2, a_3\}$, $\{a_1, a_3\}$ in formula Eq. (37) so that

$$\alpha_{a_1,a_2,a_3} M_{a_1,a_2,a_3} = M_\varnothing c_\varnothing + M_{a_1,a_2} c_{a_1,a_2} + M_{a_1,a_3} c_{a_1,a_3} + M_{a_2,a_3} c_{a_2,a_3} . \quad (42)$$

Using Eq. (39), we can write the zero-order coefficient $c_\varnothing$ as

$$\begin{aligned} c_\varnothing &= \mathbb{E}\left[\prod_{a \in \{a_1,a_2,a_3\}} (R_a - m_a)(1 - Y_a) \right] + \mu_{a_1} \mathbb{E}\left[\prod_{a \in \{a_2,a_3\}} (R_a - m_a)(1 - Y_a) \right] \\ &\quad + \mu_{a_2} \mathbb{E}\left[\prod_{a \in \{a_1,a_3\}} (R_a - m_a)(1 - Y_a) \right] + \mu_{a_3} \mathbb{E}\left[\prod_{a \in \{a_1,a_2\}} (R_a - m_a)(1 - Y_a) \right] , \\ &= \mathbb{E}\left[\prod_{a \in \{a_1,a_2,a_3\}} (R_a - m_a)(1 - Y_a) \right] \\ &\qquad + \mu_{a_1} \alpha_{a_2,a3} M_{a_2,a_3} + \mu_{a_2} \alpha_{a_1,a3} M_{a_1,a_3} + \mu_{a_3} \alpha_{a_1,a2} M_{a_1,a_2} , \end{aligned}$$

where we have utilized formula Eq. (42) for the second-order centered moments. Using Eq. (38), the second-order coefficient c_{a_1,a_2} can be written as

$$c_{a_1,a_2} = \gamma_{a_1,a_2,a_3;a_1,a_2} - \alpha_{a_1,a_2,a_3} \mu_{a_3} .$$

with

$$\gamma_{a_1,a_2,a_3;a_1,a_2} = \mu_{a_3} \mathbb{E}\left[Y_{a_1} Y_{a_2} (1 - Y_{a_3})\right] + \mathbb{E}\left[Y_{a_1} Y_{a_2} (R_{a_3} - m_{a_3})(1 - Y_{a_3})\right] .$$

Utilizing the definition of α_B given in Eq. (32), we have

$$\begin{aligned} c_{a_1,a_2} &= \mathbb{E}\left[Y_{a_1} Y_{a_2} (R_{a_3} - m_{a_3})(1 - Y_{a_3})\right] + \mu_{a_3} \left(\mathbb{E}\left[Y_{a_1} Y_{a_2} (1 - Y_{a_3})\right] - \alpha_{a_1,a_2,a_3} \right) , \\ &= \mathbb{E}\left[Y_{a_1} Y_{a_2} (R_{a_3} - m_{a_3})(1 - Y_{a_3})\right] - \mu_{a_3} \left(\frac{1}{b\tau_{a_3}} + \alpha_{a_1,a_2} \right) . \end{aligned}$$

Combining all the above results in Eq. (42) finally leads to the compact expression

$$\begin{aligned}
&\alpha_{a_1,a_2,a_3} M_{a_1,a_2,a_3} = \\
&\qquad \mathbb{E}\left[\left(R_{a_1}-m_{a_1}\right)\left(1-Y_{a_1}\right)\left(R_{a_2}-m_{a_2}\right)\left(1-Y_{a_2}\right)\left(R_{a_3}-m_{a_3}\right)\left(1-Y_{a_3}\right)\right] \\
&\qquad + \mathbb{E}\left[\left(R_{a_3}-m_{a_3}\right)\left(1-Y_{a_3}\right)\left(Y_{a_1}Y_{a_2}-1\right)\right] M_{a_1,a_2} \\
&\qquad + \mathbb{E}\left[\left(R_{a_2}-m_{a_2}\right)\left(1-Y_{a_2}\right)\left(Y_{a_1}Y_{a_3}-1\right)\right] M_{a_1,a_3} \\
&\qquad + \mathbb{E}\left[\left(R_{a_1}-m_{a_1}\right)\left(1-Y_{a_1}\right)\left(Y_{a_2}Y_{a_3}-1\right)\right] M_{a_2,a_3} \, .
\end{aligned}$$

Appendix 6. Event rates definitions and calculations. Let us consider a set of neurons B. By definition, each neuron $a \in B$ experiences a synaptic event whenever $W_a = W_{\mathrm{e},a} + W_{\mathrm{i},a} > 0$, where $W_{\mathrm{e},a}$ and $W_{\mathrm{i},a}$ denotes the random excitatory and inhibitory jumps delivered to neuron a. Let us denote the overall rate of synaptic events collectively experienced by the set of neurons B by b_B. Because neurons are allowed to receive synchronous, the rate b_B is generally strictly less than the sum of the event rates b_a experienced by each neuron $a \in B$ individually. However, the overall rate b_B can be expressed in terms of the individual rates b_a at the cost of introducing auxiliary conditional probabilities. To see this, one needs to first determine the event rate b_B in terms of the event rate of all subset $A \subsetneq B$. This can be done by remarking that

$$\begin{aligned}
b_B &= b_B \mathbb{E}\left[\mathbb{1}_{\{\sum_{a\in B} W_a > 0\}}\right] , \\
&= b_B \mathbb{E}\left[\sum_{\varnothing \subsetneq A \subset B} (-1)^{|A|-1} \prod_{a\in A} \mathbb{1}_{\{W_a>0\}}\right] , \\
&= \sum_{\varnothing \subsetneq A \subset B} (-1)^{|A|-1} b_B \mathbb{E}\left[\prod_{a\in A} \mathbb{1}_{\{W_a>0\}}\right] , \\
&= \sum_{\varnothing \subsetneq A \subset B} (-1)^{|A|-1} b_A \mathbb{E}\left[\prod_{a\in A} \mathbb{1}_{\{W_a>0\}} \middle| \sum_{a\in B} W_a > 0\right] . \qquad (43)
\end{aligned}$$

Accordingly, let us denote the probability that all neurons $a \in A$ spikes given that a synaptic event occurs for B (i.e. at least one neuron $a \in B$ spikes) by

$$q_{A,B} = \mathbb{E}\left[\prod_{a\in A} \mathbb{1}_{\{W_a>0\}} \middle| \sum_{a\in B} W_a > 0\right] ,$$

and for simplicity, let us also denote $q_B = q_{B,B}$. The above probabilities are the auxiliary conditional probabilities required to specify b_B. Indeed, solving for b_B in Eq.(43), one obtains

$$b_B = \frac{1}{1+(-1)^{|B|} q_B} \sum_{\varnothing \subsetneq A \subsetneq B} (-1)^{|A|-1} b_A q_{A,B} \, ,$$

which upon iterated use yield an explicit formula specifying b_B in terms of b_a, $a \in B$:

$$b_B = \sum_{a\in B} b_a \sum_{m=1}^{|B|} \sum_{\{a\}=B_1 \subsetneq \ldots \subsetneq B_m = B} (-1)^{\sum_{k=1}^{m} |B_k| - m} \prod_{k=1}^{m} \left(\frac{q_{B_k,B_{k-1}}}{1+(-1)^{|B_m|} q_{B_m}} \right) .$$

The above formula and its derivation is perhaps easier to grasp for the case when B represents a pair of neurons, which we discuss now. To this end, let us denote

$W_1 = W_{\mathrm{e},1} + W_{\mathrm{i},1}$ and $W_2 = W_{\mathrm{e},2} + W_{\mathrm{i},2}$, then we have

$$\begin{aligned} b_{12} &= b_{12}\mathbb{E}\left[\mathbb{1}_{\{W_1+W_2>0\}}\right] , \\ &= b_{12}\mathbb{E}\left[\mathbb{1}_{\{W_1>0\}} + \mathbb{1}_{\{W_2>0\}} - \mathbb{1}_{\{W_1>0,W_2>0\}}\right] , \\ &= b_1 + b_2 - b_{12}q_{12} , \end{aligned}$$

where we have defined $q_{12} = \mathbb{P}\left[W_1 > 0, W_2 > 0 \,|\, W_1 + W_2 > 0\right]$. This directly implies that

$$b_{12} = \frac{b_1 + b_2}{1 + q_{12}} .$$

One can thus check that when inputs to neuron 1 and neuron 2 are independent, one has $q_{12} = 0$, so that one obtains the upper bound $b_{12} = b_1 + b_2$ as expected. By contrast, when neurons are perfectly synchronous, so that the neuron with minimum input rate $\min(b_1, b_2)$, only receives inputs when the neurons with maximum input rate $\max(b_1, b_2)$ also receives an input, one has $q_{12} = \min(b_1, b_2)/\max(b_1, b_2)$. Thus, one obtains the lower bound

$$b_{12} = \frac{b_1 + b_2}{1 + \min(b_1, b_2)/\max(b_1, b_2)} = \max(b_1, b_2) ,$$

as expected.
