# Supplementary material for: Subthreshold moment analysis of neuronal populations driven by synchronous synaptic inputs
Source: PLoS Comput Biol. 2025 Oct 31;21(10):e1013645. doi: 10.1371/journal.pcbi.1013645 (PMC12629500; doi:10.1371/journal.pcbi.1013645)
Supplement: Attachment — Submitted filename: ResponseToReviewers.pdf [file pcbi.1013645.s002.pdf]

# Response to the reviewers of the manuscript titled "Subthreshold moment analysis of neuronal populations driven by synchronous synaptic inputs" (PCOMPBIOL-D-25-00525)

Dear Editor,

Thank you for taking the time to review our manuscript titled "Subthreshold moment analysis of neuronal populations driven by synchronous synaptic inputs". This letter is a response to the reviewers's comments and a description of the changes to the manuscript. We appreciate the time and dedication that the reviewers spent on our manuscript.

Sincerely,

Logan Becker, François Baccelli, Thibaud Taillefumier

## Response to Reviewer 1

One of the main conclusions (as in their previous paper) is that only a small number of asynchronous inputs can explain the variability in membrane dynamics observed in vivo. The authors therefore claim that with physiologically realistic synapse counts synchronous inputs are more consistent with the data. However, synaptic weights vary greatly in size, and are approximately lognormally distributed in pyramidal cells in cortex (and elsewhere). Not taking into account that synaptic weights are broadly distributed limits the conclusions that can be drawn from the model. This should be addressed up front. I think that the approach is general enough to allow for such distributions.

As noted by the reviewer, an apparent limitation of our analysis is the assumption that there is a typical synaptic size. Taken literally, this assumption implies that conductance variability primarily stems from the variable numbers of coactivating synapses, and thus from synchrony. This is certainly an oversimplification as synapses exhibit heterogeneity [1], which likely plays a role in shaping neural variability [2]. Distinguishing between heterogeneity and synchrony contributions, however, is a fundamentally ambiguous task [3]. For instance, consider $K$ inputs with identical weight $w$ and identical spiking rate $r$. Such inputs are instantaneously synchronous if $p(k)$, the probability that $k$ of them coactivate, is

not concentrated on $k = 1$ but also distributed on values $k > 1$. It is not too hard to realize that such a synchronous model is indistinguishable from considering $K$ independent inputs with heterogeneous weights $w$, $2w$, ..., $Kw$ and rates $Krp(k)$. This ambiguity mirrors the ambiguity of interpreting whether EPSP results from a single or multiple coincidental synaptic activations. That said, the interpretation of our results in terms of synchrony rather than heterogeneity is supported by recent experimental evidence that cortical response selectivity derives from strength in numbers of synapses, rather than difference in synaptic weights [4]. More importantly, and as noted by the reviewer, our framework is general enough to account for the impact of heterogeneities. Actually, all our analytical results are derived assuming synaptic weights heterogeneities in our manuscript. It is only when we discuss these results that we assume a typical synaptic weight for the sake of clarity. In any case, we can use our framework to gauge the contribution of synaptic heterogeneity to neuronal covariability and compare it to the contribution of input synchrony. Performing such an analysis reveals that in the absence of synchrony, the heterogeneity contribution to variability is primarily controlled by the coefficient of variation (CV) of the synaptic weights. To see why, let us consider the case of asynchronous heterogeneous excitatory inputs but with fixed rate $r$ for simplicity. The subthreshold variance then reads:

$$\mathbb{V}[V] = \frac{r \sum_{k=1}^{K} w_k^2 (V_e - \mathbb{E}[V])^2}{2(1/\tau + r \sum_{i=1}^{K} w_k)} \,.$$

Identifying the typical value of synaptic weights to the mean synaptic weights, we then have:

$$\sum_{k=1}^{K} w_k^2 = K\mathbb{E}[W^2] = K(\mathbb{E}[W]^2 + \mathbb{V}[W]) = K\mathbb{E}[w]^2(1 + \mathrm{CV}^2[W]) \,.$$

This shows that the contribution of heterogeneity to variability is mediated by the squared coefficient of variation of the weights, which we denote $\mathrm{CV}^2[W]$. This coefficient has been inferred from EPSP amplitude measurements in several experimental studies. These measurements, including those reported in the studies [5, 6] cited in [1] indicate that the CV of EPSP amplitudes is smaller than 1 (about 0.4 in most studies). This supports that synaptic weight heterogeneity can at most double subthreshold variability, which is not enough to achieve realistic voltage variance values.

This is by contrast with the synchrony contribution. Indeed, formally, the term $\mathrm{CV}[W]$ plays the same role as the term $\rho(K-1)$, where $\rho$ denotes the input spiking correlation. Given 1000 inputs with very weak spiking correlation $\rho = 0.01$ (low end of in-vivo measurements), we expect $\rho(K-1)$ to be about 10, so that variability is increased by at least an order of magnitude in the presence of synchrony. This supports our hypothesis of synchrony being the main driver of variability.

In keeping with the reviewer's recommendation, our revised manuscript now discusses the role of synaptic heterogeneity along the lines explained above. Specifically, we have briefly discussed synaptic heterogeneity upfront in the section about biophysical parameters and have included a more detailed paragraph about synaptic heterogeneity in the discussion.

Physiological spike trains often exhibit structured temporal correlations (bursting, refractoriness), which could influence neuronal variability. The authors note this, but it would be

good to note that this temporal variability can also impact the results. Also, if synchronous spikes are not precisely aligned in time, I would assume that stronger correlations are needed to recover the same results. On the other hand, the temporal dynamics of synaptic inputs will affect the conclusions as well.

We agree with the reviewer's comment and confirm his intuition that stronger correlations are needed when spikes are not precisely aligned. Actually, our manuscript includes an explanation for this intuition in the discussion section titled "More realistic form of synchrony". Specifically, we explain how our results remain predictive of variability measurements when inputs are first generated to be highly temporally synchronous (with much higher correlations than measured experimentally) and then independently jittered with some noise functions (this jittering erases the high degree of correlations). The resulting jittered synchrony produces a form of synchrony that is more realistic as it mimics the temporal structure of synchrony observed in vivo, as reported in [7]. In particular, spiking correlation values become dependent on the bin size in which spikes are counted. Although an exact treatment of this more realistic form of synchrony was not possible, we found that our formula applies if one uses spiking correlation coefficients measured for bin sizes of duration similar to the membrane cell passive time constant.

We discussed this point in detail in our prior publication in PRX. For the sake of completeness, we reproduce the key points of that discussion in the following:

One of the features of instantaneous spiking correlations is that for such input models, empirical estimates of the spiking correlations are independent of the timescale or bin size over which spikes are counted, as shown in Fig. R1A (blue line). This is a potential problem because as pointed out by the reviewer, spiking correlations have been measured to vanish on small timescales in experimental recordings. Following the reviewer's suggestion, spike jittering precisely synchronous spikes over time windows of duration T leads to a general decrease in spiking correlations at timescales smaller or of the order of T, with spiking correlations that vanish in the limit of small timescales (red, yellow and purple lines). The key observation, however, is that such jittering also substantially decreases the level of spiking correlations over 25ms windows (vertical dashed line in Fig. R1A). For large jittering timescale, such a decrease conflicts with our requirement that correlations lie within the range 0.01-0.04 at 25ms. To correct for this decline at 25 ms, the instantaneous spiking correlation of the unjitterred correlated input model may be increased. To make appropriate comparisons the jittering procedure should consist of adjusting the level of instantaneous spiking correlation necessary to achieve the range 0.01-0.04 over the 25ms timescale. Adopting a jittering timescale of 50ms, we show that spiking correlation coefficients of about 0.03 are achieved in bins of 25ms for unjittered spiking inputs with instantaneous spiking correlation between 0.2 and 0.3. Note that for bins of very long size, the spiking correlation will saturate at values of 0.2 or 0.3, i.e, at similar levels as those reported in [a].

Next, we validate that our model makes realistic predictions about the subthreshold variability. To do so, we simulate AONCB neurons in response to realistic, temporally structured, correlated inputs. In Fig. R1C, we show that the corresponding stationary voltage distribution (red trace and histogram) closely follows the distribution that would be obtained by assuming instantaneous spiking correlations $\rho_0$ equal to the 25ms spike-count correlation

of the realistic inputs (blue trace and histogram). Furthermore, we empirically justify the choice of the instantaneous correlation parameter $\rho_0$ as the 25ms spike-count correlation of the realistic input model. Specifically, in Fig. R1D, we consider 3 temporally structured inputs obtained from the same instantaneous synchronous inputs but for various jittering. Jittering at larger timescale reduces synchrony and thus decreases voltage variance (vertical dashed lines). We then compare the voltage variance with perfectly synchronous approximation obtained by matching spike-count correlation at various time scale (our choice is to match at 25ms). Fig. R1D shows that matching at increasing timescale yields higher variance and matching around 25ms offers good approximations (grey square where variances are about the same). Although, we do not have an analytical argument to support this empirical finding, we checked that this close correspondence between stationary distribution with realistic correlated inputs and exactly synchronous inputs is maintained for varying input conditions. For instance, Fig. R2 shows that realistic levels of subthreshold variability can still be inferred from our analysis in the low drive regime.

The authors also do not model negative correlations which could significantly impact dynamics. What would be the effect of negative correlations which are also observed often.

The reviewer is correct to note that we do not consider negative correlations. There are essentially three reasons for this.

First, in a stationary framework, instantaneous spiking correlations must be nonnegative from a mathematical standpoint. This is because of the representation of spike trains as randomly distributed, $\{0, 1\}$-values, multidimensional arrays, which are invariant by time-shifts. Although negative correlations are possible in time bins of nonzero durations, only nonnegative correlations can persist in the limit of vanishingly small time bins, which corresponds to our instantaneous form of synchrony. Including negative correlations would require considering more realistic forms of synchrony, which may be modeled via doubly stochastic processes instead of Poisson point processes. However, a treatment of this case is not possible via a stationary PASTA analysis.

Second, most measurements of spiking pairwise correlations, especially when evaluated over large bin sizes, are positive [7, 8]. The main caveat to this observation lies in the fact that aggregate excitatory and inhibitory spiking activities tend to be anticorrelated at small time scales. This stems from the fact that inhibition is known to lag excitation by a few milliseconds in cortex [9]. Because such negative correlations arise from transient dynamics over timescales shorter than the membrane timescales, we do not expect them to impact the stationary response of individual cells.

Third, the formulas that we derived under assumptions of positive spiking correlations may be applied to negative correlations when considered in the small-weight approximation. One can simply allow for negative spiking correlation coefficients, independent of whether this corresponds to an actual statistical model for synaptic inputs. However, we have no rigorous or qualitative arguments to justify the validity of these formulas when negative correlations are allowed. Preliminary simulation results support that our formula can predict variability in the presence of negative correlations only if these take place in between clusters of positively correlated inputs and if these negative correlations are small compared to the positive correlations within clusters.

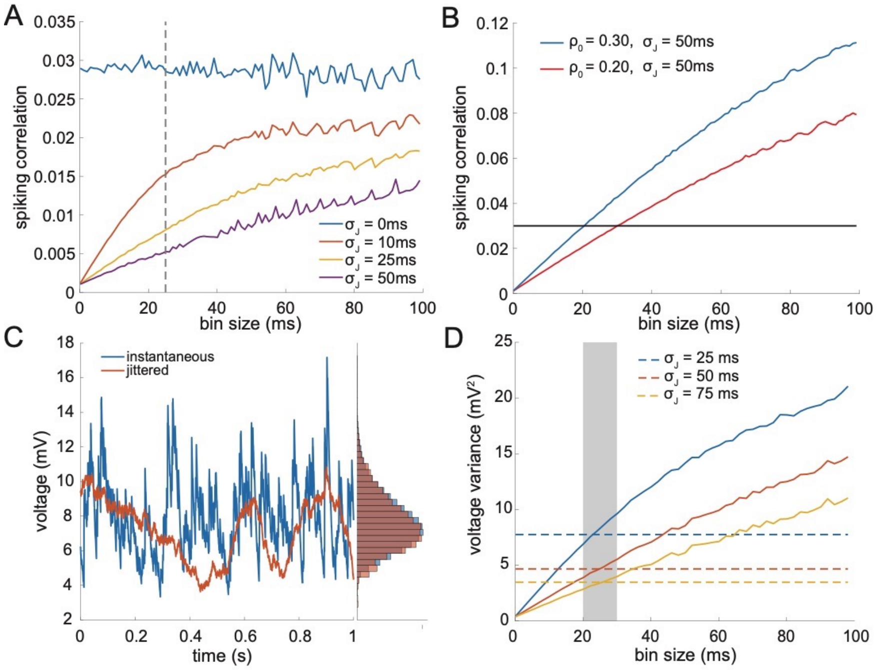


Figure R1: A. Effect of jittering spike times from our synchronous input model via independent Gaussian centered jitters with varied standard deviation $\sigma_j$: in the absence of jitter, spiking correlation is independent of the size of the time bins used to count spikes (blue trace). Jittering with larger time scales increasingly decreases spiking correlation for all bin sizes, and spiking correlations vanish in the limit of small bin sizes. B. Given a jitter standard deviation of 50ms, one obtains spike-count correlation of 0.03 in 25ms bins by jittering of 50ms standard deviation a synchronous input with a comparatively large instantaneous correlation of $\sigma_o = 0.2 - 0.3$. C. Comparison of voltage trace obtained with instantaneous synchronous input (blue) and jittered correlated inputs (red) for $\sigma_j = 50$ms. Both types of input are chosen so that they yield the same spiking correlation of 0.03 in bin size of 25ms. The stationary distributions are close to identical leading to less than 1% error in the variance estimates. D. Comparison between the voltage variances of AONCB neurons driven by realistic synchronous inputs with various jitter (dashed line) with voltage variances of AONCB neurons driven by instantaneously synchronous approximations obtained by matching spiking correlation at various timescale for bin sizes (solid line). Different instantaneous approximations are obtained for different bin sizes T by setting the instantaneous correlation parameter $\sigma_o$ equal to the spiking correlation of the jittered inputs measured at that bin size T. Good approximations are consistently obtained by instantaneous correlation parameter measured around the 25ms timescale (grey column). Other parameters: $r_e = 10$Hz, $K_e = 1000$, $w_e = 10 - 3$.

For all these reasons, we would prefer not to discuss negative correlations in the manuscript. In fact, we are currently developing nonstationary methods, which excludes the use of the PASTA principle, to study the impact of negative correlations.

There are many other ways to model synchronous inputs - for instance see the work of Krumin and Shoham, Kuhnn, Aertsen and Rotter, and Trousdale, Shea-Brown and Josic. These approaches also allow for jittering. It would be good to discuss briefly how these previous models are related to the present model - and in particular whether approaches similar to

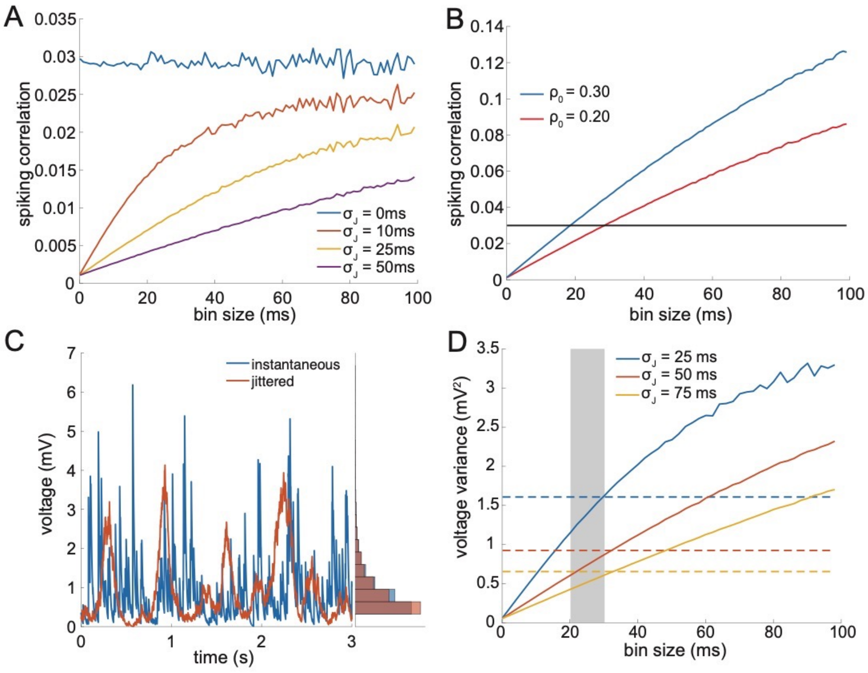


Figure R2: Same as Figure 1 but for low rates: $r_e$ =1Hz. The variance estimated from both distribution in C. differ by about 1

those proposed in these previous papers could be used to get models where higher order moments can be better controlled and their impact analyzed. In particular, I assume that an approach similar to that used to define an MIP and SIP process could be used here to control the structure of higher order correlated variability across the population.

We appreciate the reviewer's advice to better link our framework to other models for synchronous spiking activity. Our revised manuscript now explicitly cites the modeling approaches referenced by the reviewer and includes a brief description about how these link to our approach. A more detailed account of these links is as follows:

The statistical model presented in [10] is a doubly stochastic one that leverages our ability to easily generate correlated Gaussian processes to simulate correlated stochastic intensities. These intensities then serve to direct individual point processes, which collectively exhibit complex correlation structures. In particular, these can exhibit non trivial temporal correlation structures. For this reason, the model considered in [11] is more general than our model, which only allows for an instantaneous form of synchrony. Actually, one can think of our model as being obtained from doubly stochastic models in the limit for which their stochastic intensities exhibit vanishing correlation times [12].

The statistical model from [13] leverages classical tricks from Poissonian analysis such as thinning to generate Poisson processes with nontrivial correlation structures. The tricks considered in [13] implicitly assume a symmetric situation whereby every neuron plays the same role. Obtaining our model by similar tricks is actually not obvious even in the symmetric situation. This is because the formal basis of our model is best understood in the context of random measures, where one can see that the spiking patterns we consider actually form

a realization of a particular clustering process, the so-called Indian buffet process (IBP) [14]. In our model, there are as many clusters as there are spiking times, and cluster sizes correspond to the number of co-spiking neurons out of a finite total population of neurons. Similar tricks as those used in [13] can be designed by exploiting our ability to exactly sample IBPs via nonparametric Markov chain MonteCarlo methods, which avoids the need to specify the infinite-dimensional measure that directs the clustering process. However, these methods are not straightforward or particularly illuminating.

The statistical model from [15] leverages our knowledge of multi-dimensional Poisson point processes to capture more realistic forms of synchrony than instantaneous ones. An important advantage of this approach is that it retains many of the tractability features of Poisson analysis, albeit in a setting where the intensity is multidimensional. Unfortunately, this setting does not allow for the application of the PASTA principle, which only applies to unidimensional Poissonian models.

Smaller Notes: Check for small grammatical errors, eg l. 218 amounts to know - amounts to knowing, l. 256 purpose - purposes, and amusing typo on l. 312

We corrected the typos. Thanks for the careful reading.

Define the acronym PASTA, unless I missed it

The first mention of the PASTA principle occurs in the following sentence (line 314-315 of the originally submitted manuscript): "In this work, we utilize one such result, the so-called PASTA principle from queuing theory, which stands for "Poisson arrivals see time averages" [32, 33]". However, to make the meaning of the acronym more apparent, we change the section title about the PASTA principle to "PASTA (Poisson Arrivals See Time Averages) principle".

I did not check all the derivations, although the results seem plausible. It would be good if the most important analytical expressions are checked against simulations. This is not necessary for all expressions.

We checked our calculations or are confident that these are correct. We also include a more detailed comparison to numerical simulations in Fig. R3 in our response.

# Response to Reviewer 2

Most of the figures in the paper present results of Monte Carlo simulations, however some key details of the implementation of these simulations are not clearly specified. While the effect of input events on the membrane potential is thoroughly explained (i.e. the Marcus dynamics), it is unclear how input events with desired statistics are generated. In particular, the authors must include a clear description of how the $(\{X_{e,k}\}_{1\leq k\leq K_e}, \{X_{i,l}\}_{1\leq l\leq K_i})$ are chosen for each event time, in order to ensure the correct statistics for the inputs.

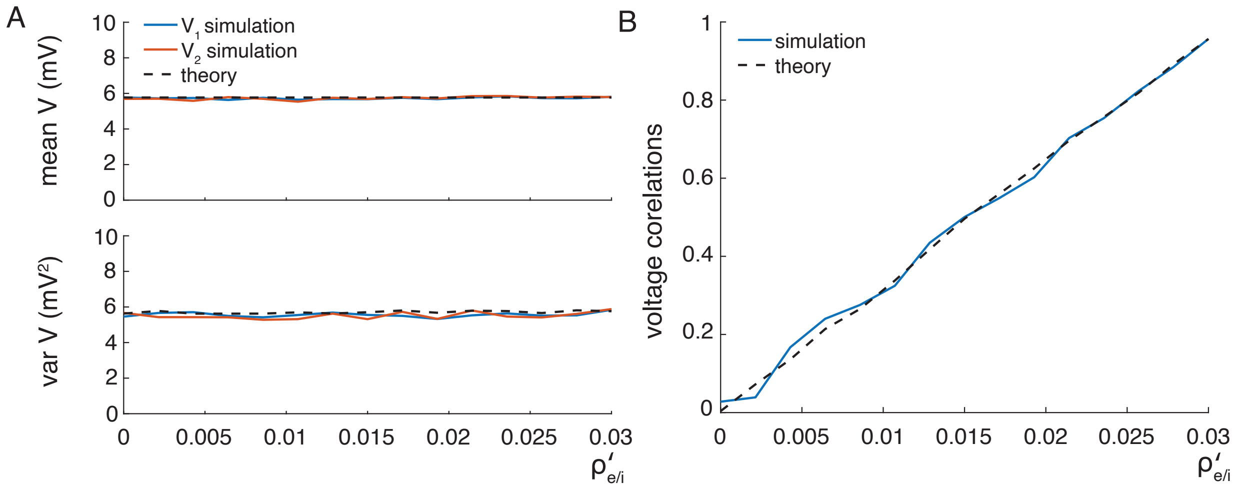


Figure R3: Comparison of Monte Carlo simulations to theoretical predictions with increasing excitatory ($\rho'_e$) and inhibitory cross-neuron input correlations ($\rho'_i$). (a, top) Mean subthreshold voltage of two neurons and theory estimates as a function of increasing cross-neuron input correlation $\rho'_e$. (A, bottom) Voltage variance under the same conditions as (A, top). (B) Average subthreshold voltage correlation and theory estimates between neurons as a function of increasing $\rho'_e$ and $\rho'_i$. In call cases we used moderate weights with $w_i = 4w_e$ and $K_e = 1000$, $r_e = 10$ Hz, $\rho_e = 0.03$, $K_i = 250$, $r_i = 10$ Hz, $\rho_i = 0.03$, and $\rho_{ei} = \rho'_{ei} = 0$.

The reviewer is correct to note that our initial submission did not include implementation details for generating correlated inputs in the Monte Carlo simulations. To address this, we have added a subsection titled "Numerical simulation methods" in the Methods section. There, we describe in considerable detail how we use Beta-Gaussian copulas to generate spiking inputs with specified within- and cross-neuron correlations. This method is flexible and scales to any number of input pools or output neurons. Examples of presynaptic inputs for a single population, two independent populations, and cross-population interactions, and their agreement with theory, are shown in Fig. R4. Please note that we also include code used to generate correlated inputs throughout our paper as available in our project GitHub.

Moreover, for the simulation results shown in Figure 11, it is unclear how the external uncorrelated excitatory drive is generated and included in the simulation, especially since the parameters for the simulation are given as $\rho_e = \rho_i = 0.03$, $\rho_{ei} = 0$, $\rho'_{e/i} = 0.025$, and $\rho'_{ei} = 0$. Do these parameters specify the background with the uncorrelated drive coming from a separate population of completely uncorrelated excitatory presynaptic neurons? If so, what are the relative population sizes? These questions should be clearly answered in the main text or the caption of Figure 11.

To address the reviewer's concern, we updated the text in the subsection titled "Biophysical relevance and modeling implications" to include further details regarding the simulation parameters. In the updated text we stated that the "two neurons receive both within- and cross-neuron correlated inputs, characterized by $K_e = 1000$, $K_i = 250$, $r_e = 5$ Hz, $r_i = 10$ Hz, $w_e = 0.001$, $w_i = 0.004$, $\rho_e = 0.03$, $\rho_i = 0.03$, and $\rho'_e = \rho'_i = 0.025$. Additionally, each

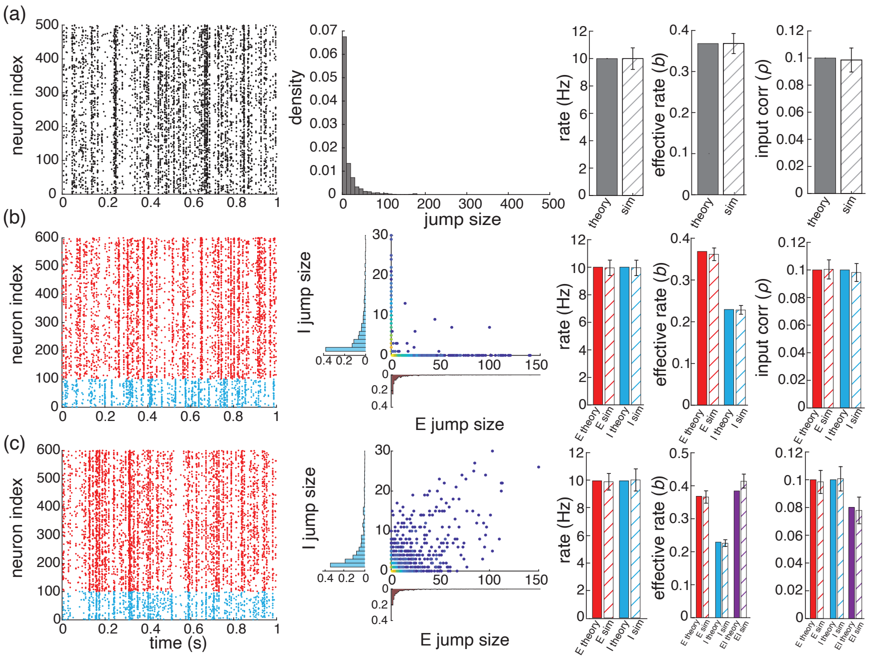


Figure R4: **Example simulations of correlated inputs using the Beta-Gaussian copula model.** Left columns shows simulated raster plot with corresponding jump size densities in middle column. Right shows strong agreement between desired/theoretical statistics (rate, effective rate $b$, and correlation $\rho$) with simulation results. Error bar shows $\pm 1$ SD. (a) Single population input with $K = 500$, $r = 10$, and $\rho = 0.1$. (b) Excitatory and inhibitory population with no cross pool correlations. $K_e = 500$, $r_e = 10$, $\rho_e = 0.1$, $K_i = 100$, $r_i = 10$, $\rho_i = 0.1$, and $\rho_{ei} = 0$. (c) Same as b. but with arbitrary cross pool correlations such that $\rho_e = \rho_i = 0.1$, and $\rho_{ei} = 0.08$ where $x_1 = 0.3$ and $x_2 = 0.004$.

neuron is driven by large, asynchronous excitatory inputs with $K_e = 250$ and $w_e = 0.01$, and varying rates, representing thalamic inputs driven by external stimuli."

There are contradictory statements regarding whether inputs are shared between different neurons. Consider the sentence "To see this, let us consider a set of neuron (sic) $A$, with cardinality denoted by $|A|$, subjected to the same set of $K_e$ excitatory and $K_i$ inhibitory synchronous inputs." (lines 281-282) This implies that inputs are shared between neurons. However, presynaptic neurons are later specified by the neuron to which they send inputs. For example, $\rho_{\alpha\beta,12,kl}$ is the correlation between $k^{\text{th}}$ input of type $\alpha$ to neuron 1 and $l^{\text{th}}$ input of type $\beta$ to neuron 2. If the inputs were shared, then the 1 and 2 indices would be completely redundant; however, we see that $\rho_{\alpha\beta,12,kl} \neq \rho_{\alpha\beta,11,kl} \neq \rho_{\alpha\beta,22,kl}$. If populations of inputs do not overlap between the different neurons, then language suggesting they do must be removed.

We apologize for the confusion and clarify our notations and their meaning in the revised manuscript. Specifically, we make clear that we consider a pool of inputs on their own, separately from which neurons they may connect to. As a result, and in agreement with the reviewer's comment, the correlation coefficients do not need to include any indexation with respect to downstream neurons' indices. We reviewed all our notations to dispel any inconsistency.

In Figure 5, the color bars are somewhat misleading. Since the goal is to compare voltage variance between the six different scenarios, the six color bars should all use the same scale. Alternatively, a separate plot could be included in the figure comparing the variances between all six panels

The reviewer raises a valid concern regarding the scaling of the heatmaps used to illustrate how changes in input affect voltage variance. Our objective in Figure 5 was to summarize how the variance evolves with input rate and how it scales with input correlations and synaptic weights. While using different scales for each subplot may initially seem confusing, we found that it provides the clearest representation of the data. When applying a uniform scale across all subplots, the large disparity between the moderate-weight asynchronous and large-weight synchronous input conditions leads to significant visual compression of important features, as shown in Figure R5. As an alternative, we considered applying a logarithmic scale to preserve the full range of values, illustrated in Figure R6. However, while this approach maintains consistent scaling, it fails to capture qualitative differences in structure as effectively as standard scaling with varying axis limits.

If the reviewer prefers, we are prepared to revise the figure using either uniform linear or logarithmic scaling for all subplots.

There are issues with the equation referencing, especially within the Supplementary Information. For example, there are several references to Eq. (45), but there are only 44 numbered equations in the manuscript.

Apologies for the confusion. We reviewed our equation referencing and made sure that

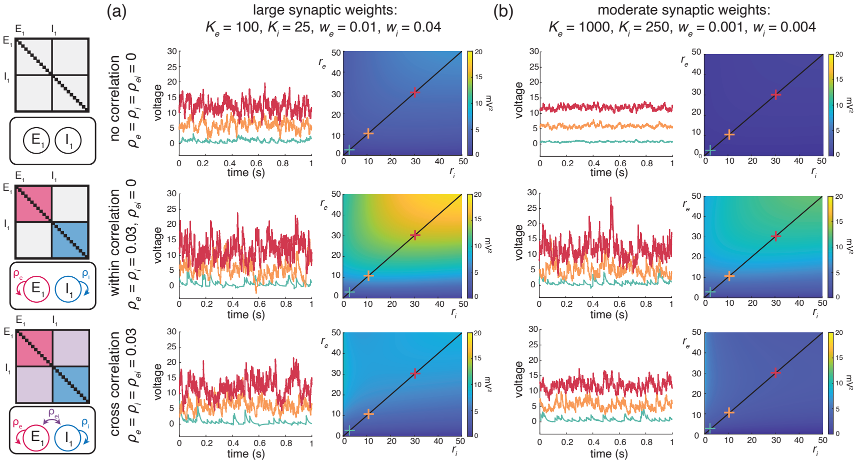


Figure R5: Figure 5 identical linear scaling across subplots

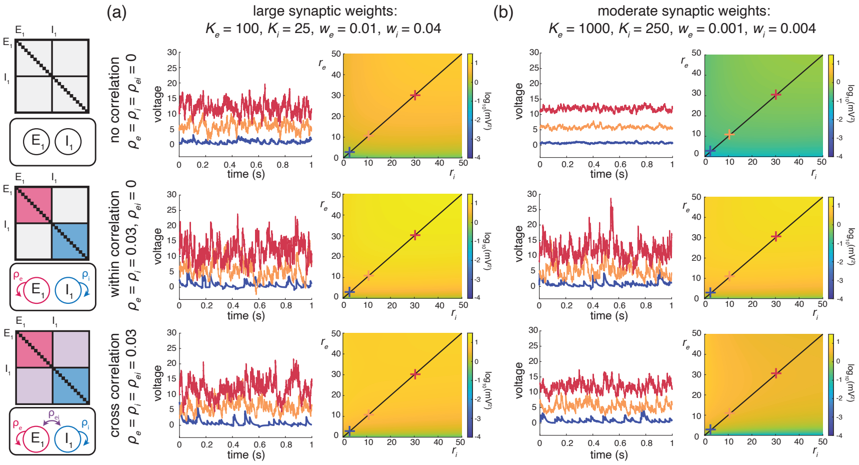


Figure R6: Figure 5 identical $\log_{10}$ scaling across subplots

each equation was properly referenced.

There are many typos within the text that should be cleaned up. An important example is that the multiset defined on line 338 is called $B$, but seems to be referred to as $A_n$ in the subsequent equations. Also, in Eq. (27) the $a \subset B$ in the first square bracket should be $a \subset C$ and "time-shit" on line 312 should be "time-shift". We strongly recommend a thorough proofreading of the text to find and correct all typos.

We acknowledge some unfortunate typos. We have proofread the manuscript many times and believe that there are no typos left.

# Response to Reviewer 3

The model studied here is simplistic. Besides the assumption of linearity, which is both a strong limitation and a necessity to be able to carry out analytics, I am worried about the absence of adequate treatment of the instability threshold. This threshold, leading to the production of an action potential, lies about 15 mV above the resting potential. If I look at the numerical plots in the paper, e.g. in Figures 5 and 8, it seems that such values are not extremely unlikely (and they should not be for a model supposed to represent a population of spiking neurons!). The authors also reports (line 396) the existence of electrophysiology experiments estimating the standard deviation of the voltage to be 3 mV, which makes the 15mV gap not so wide. It is therefore important to take into account the reset mechanism for the voltage, which will affects its statistics. This is equivalent to a first passage-time problem, which can be (and was already) handled in the case of the linear integrate-and-fire neuron model.

We agree with the reviewer's comment that neglecting the spike-generating mechanism as a source of neural variability may be an important limitation. Most diffusion-based approaches model spike generation via the integrate-and-fire mechanism, whereby the membrane voltages reset to fixed value upon reaching a spike-initiation. In the following, we argue that considering such a mechanism would actually obscure the analysis.

In principle, it is conceivable that the additional variability due to post-spiking resets dominates the synaptic variability in shaping subthreshold variability. In such a case, a large overall subthreshold variability, consistent with physiological reports, may even be achieved for low synaptic variability. However, this possibility is unrealistic as this would correspond to voltage traces that are quasi deterministic trajectories conditionally to the output spiking times. This is contrary to experimental observations, which suggests that spiking reset is not a major determinant of variability. In that respect, we stress that some of the limitations due to the spike-generating mechanism can be circumvented experimentally by studying the spontaneous and evoked subthreshold responses in artificially silenced, through the injection of hyperpolarizing current neurons. It is also worth noting that the experimental variance estimates that we use are extracted from voltage traces where spiking events have been excluded, so that the large variability reported can be attributed to synaptic inputs and not to the neuron's own spiking.

We have also numerically studied the effect of including an integrate-and-fire mechanism and found that it tends to produce unrealistic skewness. This is because in the driven regime, when the mean is close to the threshold, a hard threshold/reset rule leads to voltage distribution with fat left tails. This follows from resetting far away from the mean voltage, which happens to be close to the threshold values in the strongly evoked regime. This form of skewness is not observed in vivo. Actually, in-vivo measurements revealed that the distribution becomes more Gaussian (near zero skewness) in the driven regime. Consistent with these observations, in-vivo measurements show the voltage very quickly set around its mean value post spiking, so that reset only moderately impacts subthreshold variability.

That said, integrating an integrate-and-fire mechanism in our analysis is possible. However, in this case, applying the PASTA principle would produce fixed-point equations that take the form of Volterra integral equations. For being in general analytically intractable,

this approach is extremely unlikely to produce interpretable formulas.

The authors rightly emphasize the presence of strong correlations between nearby neurons in brain circuits. But they do not consider a major source for such correlations, that is, the presence of gap junctions. I think their analysis could be extended to include these interactions, as the equations remain linear, at the price of increasing the dimensionality of the tracked potential population (following the dynamics of a single neuron is not sufficient any longer).

We thank the reviewer for their insight. It is true that our framework extends to include the impact of gap junctions. Following on the author's comment, we have added a section in the discussion about how our analysis changes in the presence of electrical coupling between cells. For the sake of conciseness, we developed our arguments for a pair of electrically coupled neurons and showed how such coupling impacts the expression of their stationary mean voltages. Although straightforward, the involved calculations are substantially more tedious than those already presented, and it is hard to find compact, interpretable expressions, except for the mean case. Therefore, we only discuss the analytical treatment of this case in the manuscript.

More generally, further analysis reveals that the effect of electrical coupling is in keeping with intuition: it brings the mean voltage of the two neurons closer together, it marginally reduces individual variability by averaging, and it increases voltage correlation for obvious reasons. In support of these findings, we provide additional simulation results shown in Fig. R7. We do not include the figure in the manuscript as the results do not reveal any surprising findings.

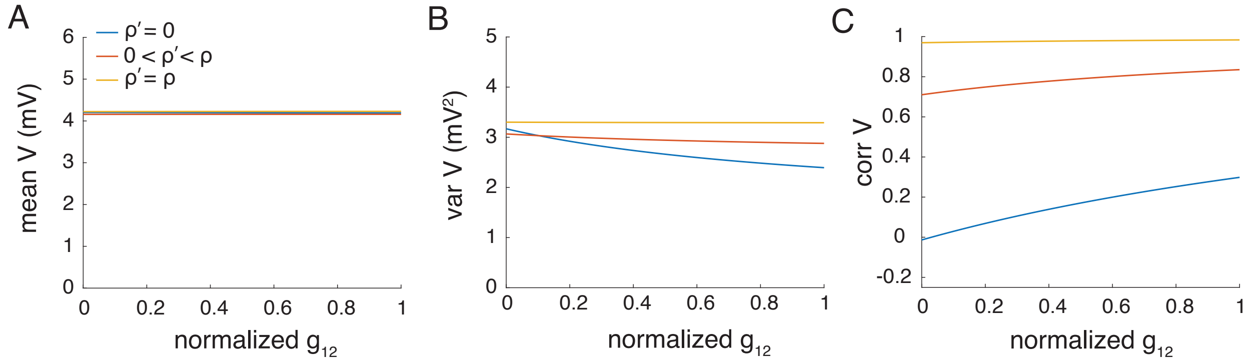


Figure R7: Effects of gap junctions on subthreshold voltage correlations. (A) Mean voltage as a function of increasing gap junction conductance ($g_{12}$) for three conditions: no cross-neuron input correlations (blue), weak cross-neuron correlations (red), and strong cross-neuron correlations (yellow). Max gap junction conductance set to over 2 times the average excitatory synaptic conductance. (B) Same as (A), but for voltage variance. (C) Same as (A), but for voltage correlations. In all cases, moderate synaptic weights were used $w_i = 4w_e$ and with $K_e = 1000$, $r_e = 5$ Hz, $\rho_e = 0.03$; $K_i = 250$, $r_i = 5$ Hz, $\rho_i = 0.03$; and no excitatory-inhibitory correlations ($\rho_{ei} = \rho'_{ei} = 0$).

My understanding of an article in PLoS Computational Biology is that detailed comparison

to experiments should be included and that a purely mathematical analysis of a model is not sufficient (this is ultimately a matter of editorial decision). The authors, at different points in the manuscript, mention some measurements and statistics, such as the values of crosscorrelations, e.g. on line 518. However, experimental time traces of membrane potentials can be obtained from intracellular recordings. It would be very interesting to push further the analysis of the model through comparison with real time traces and more extended statistics that can be extracted from those data, in particular regarding time correlations (in the stationary regime). I believe this would provide further justification for the validity of the model, which, as it is now, remains rather abstract.

We agree with the reviewer that a direct comparison with originally collected data is of paramount importance. That said, this work is the result of a collaboration between computational neuroscientists and probabilists that builds on already published experimental data. In that respect, we would like to mention that some of us have (hopefully) addressed the reviewer's comments in a manuscript that is currently under revision in another journal with a more experimental target audience. However, this manuscript is the result of a separate collaboration and we do not have permission to include the associated data in this submission.

# References


[1] György Buzsáki and Kenji Mizuseki. The log-dynamic brain: how skewed distributions affect network operations. *Nature Reviews Neuroscience*, 15(4):264–278, 2014.

[2] Ramakrishnan Iyer, Vilas Menon, Michael Buice, Christof Koch, and Stefan Mihalas. The influence of synaptic weight distribution on neuronal population dynamics. *PLoS computational biology*, 9(10):e1003248, 2013.

[3] Asohan Amarasingham, Stuart Geman, and Matthew T Harrison. Ambiguity and non-identifiability in the statistical analysis of neural codes. *Proceedings of the National Academy of Sciences*, 112(20):6455–6460, 2015.

[4] Benjamin Scholl, Connon I Thomas, Melissa A Ryan, Naomi Kamasawa, and David Fitzpatrick. Cortical response selectivity derives from strength in numbers of synapses. *Nature*, 590(7844):111–114, 2021.

[5] Sandrine Lefort, Christian Tomm, J-C Floyd Sarria, and Carl CH Petersen. The excitatory neuronal network of the c2 barrel column in mouse primary somatosensory cortex. *Neuron*, 61(2):301–316, 2009.

[6] Sen Song, Per Jesper Sjöström, Markus Reigl, Sacha Nelson, and Dmitri B Chklovskii. Highly nonrandom features of synaptic connectivity in local cortical circuits. *PLoS biology*, 3(3):e68, 2005.

[7] Matthew A Smith and Adam Kohn. Spatial and temporal scales of neuronal correlation in primary visual cortex. *Journal of Neuroscience*, 28(48):12591–12603, 2008.

[8] Matthew A Smith and Marc A Sommer. Spatial and temporal scales of neuronal correlation in visual area v4. *Journal of Neuroscience*, 33(12):5422–5432, 2013.

[9] Michael Okun and Ilan Lampl. Instantaneous correlation of excitation and inhibition during ongoing and sensory-evoked activities. *Nature neuroscience*, 11(5):535–537, 2008.

[10] Alexandre Kuhn, Ad Aertsen, and Stefan Rotter. Higher-order statistics of input ensembles and the response of simple model neurons. *Neural computation*, 15(1):67–101, 2003.

[11] Michael Krumin and Shy Shoham. Generation of spike trains with controlled auto- and cross-correlation functions. *Neural computation*, 21:1642–1664, 6 2009.

[12] Thibaud Taillefumier and Gordan Zitkovic. A scaling limit for additive functionals. 10 2024.

[13] Alexandre Kuhn, Ad Aertsen, and Stefan Rotter. Higher-order statistics of input ensembles and the response of simple model neurons. *Neural computation*, 15:67–101, 1 2003.

[14] Thomas L Griffiths, Tom Griffiths@berkeley Edu, Zoubin Ghahramani, Zoubin@eng Cam Ac Uk, and David M Blei. The indian buffet process: An introduction and review. *Journal of Machine Learning Research*, 12:1185–1224, 2011.

[15] James Trousdale, Yu Hu, Eric Shea-Brown, and Krešimir Josić. A generative spike train model with time-structured higher order correlations. *Frontiers in Computational Neuroscience*, 7:84, 6 2013.
