# Supplementary material for: Subthreshold moment analysis of neuronal populations driven by synchronous synaptic inputs
Source: PLoS Comput Biol. 2025 Oct 31;21(10):e1013645. doi: 10.1371/journal.pcbi.1013645 (PMC12629500; doi:10.1371/journal.pcbi.1013645)
Supplement: Attachment — Submitted filename: ResponseToReviewers2.pdf [file pcbi.1013645.s003.pdf]

Response to the reviewers of the manuscript titled "Subthreshold moment analysis of neuronal populations driven by synchronous synaptic inputs" (PCOMPBIOL-D-25-00525)

Dear Editor,

Thanks for reviewing our revised manuscript titled "Subthreshold moment analysis of neuronal populations driven by synchronous synaptic inputs".

We appreciate the time and dedication that the reviewers spent on our manuscript. We also appreciate the benevolence of the editor which stated that he was "not adamant on a detailed comparison to experiments (point raised by Reviewer 3), but would of course appreciate it if that was possible."

In this response, we address the remaining concerns of Reviewer 3, which we believe follows from an unfortunate misunderstanding. Specifically, (1) we provide a more thorough justification for not including the spiking mechanism in our analysis and (2) we respectfully rebut reviewers's 3 comment about the lack of direct comparison to data.

We hope that this response will be enough to bring about a favorable decision.

Sincerely,

Logan Becker, François Baccelli, Thibaud Taillefumier

Response to Reviewer 3

I thank the authors for having considered my comments and criticisms. I appreciate that they have worked out the equations for coupled neurons in the presence of gap junctions, and have inserted a paragraph in the manuscript regarding this important effect. I agree that these electrical couplings, as a major effect, enhance synchrony between cells as reported in the new paragraph.

Regarding the other points:

I am somewhat less convinced about the discussion on resetting events. In highly active areas, these events can hardly be neglected. The authors mention in their response that they

have studied "the effect of including an integrate-and-fire mechanism and found that it tends to produce unrealistic skewness." I would have liked to see these results to better assess the relevance of the effects. At the very least, I would appreciated if the authors briefly include a comment about this point in the Discussion section of the final version of the manuscript.

We understand the reviewer's concern about neglecting the reset mechanism, especially in highly active areas. There are, however, several reasons why we feel this neglect is justified in the specific context of our study:

First, the goal of our analysis is to demonstrate the key role that synaptic input synchrony plays in shaping the variability of membrane voltage traces observed *in vivo*. In this spirit, our approach focuses on the variability that arises from synaptic input integrations, excluding the influence of the cell's own spiking. Thus, although neglecting the spiking mechanism allows for our exact analytical treatment, it is also well motivated by the question considered in this manuscript.

Second, and consistent with our approach, the estimates of voltage variance and skewness that we use in the manuscript (some of which were also used in [12]) were measured for voltage traces that exclude short time windows around action potentials [3, 4]. In other words, the experimental measurements that we use to compare with our modeling predictions exclude the influence of the cell's own spiking.

Third, the inclusion of a hard post-spiking reset as is traditional in many computational studies, is problematic when modeling subthreshold variability. Indeed, integrate-and-fire neurons that reset at a fixed value, close to their leak reversal potential, and thus away from their spiking threshold, exhibit negative skewness in the driven regime. This is contrary to experimental *in vivo* observations, which reveal that neurons tend to reset to a voltage value close to their mean trend (which depends on the driving condition), as opposed to a fixed reset value. Reset rule to the mean yields similar results as when neglecting the influence of the cell's own spiking on subthreshold variability. In particular, we confirm in Fig. R1 that by contrast with reset rules to a fixed value, a reset rule to the mean produces a realistic skewness response which never become negative, as experimentally reported.

Fourth, modeling voltage traces in nonspiking neurons is of experimental relevance. This is because *in-vivo* voltage measurements can be made in neurons whose spiking is precluded by hyperpolarizing current injections as in [13]. We recently gained access to such recordings via a separate collaboration with experimentalists and are in the process of analyzing this data. This analysis will be part of another work, with distinct authors. That said, we are willing to share preliminary results in this response, hoping that these will address Reviewer 3's concerns about the relevance of our approach to experimental results. These preliminary results are shown in Fig. R2.

Fifth, it is worth noting that even in the presence of spikes, voltage variability is often attributed to properties of the synaptic drive rather than the cell's own spiking. For instance, reference [7] states in text that "measures of correlated membrane potential fluctuations are entirely dominated by subthreshold activity because APs in layer 2/3 neurons of mouse barrel cortex occur at low rates both during quiet wakefulness and active whisking periods." Even more, reference [6] specifically tested for the role of spiking in promoting voltage crosscorrelation in the visual cortex of cats and found that "this synchronization

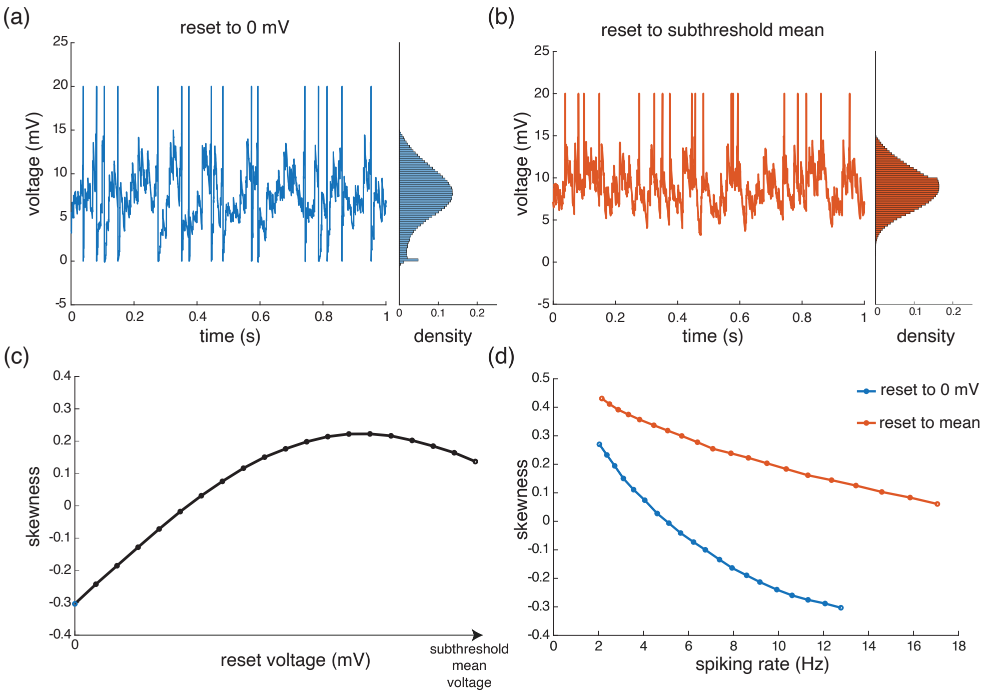

Figure R1: **Effects of Integrate-and-Fire reset on voltage skewness under correlated inputs.** (*a*) Example voltage trace and corresponding voltage histogram when the reset is set to the leak potential, $V_{\mathrm{L}} = 0$. Voltage spikes are artificially added and excluded from the histogram. The neuron receives both excitatory and inhibitory feedforward inputs with parameters $K_{\mathrm{e}} = 1000$, $r_{\mathrm{e}} = 10\mathrm{Hz}$, $K_{\mathrm{i}} = 250$, $r_{\mathrm{i}} = 5\mathrm{Hz}$, $w_{\mathrm{i}} = 4w_{\mathrm{e}}$ and threshold $V_{\mathrm{T}} = 15\mathrm{mV}$. (*b*) Same as (*a*), but with reset to the theoretical subthreshold mean predicted from the input parameters. (*c*) Dependence of voltage skewness on reset voltage, ranging from the leak potential (0 mV) to the subthreshold mean. (*d*) Voltage skewness as input strength increases (via increased synaptic weights) for resets to 0 mV (blue) and to the subthreshold mean (orange).

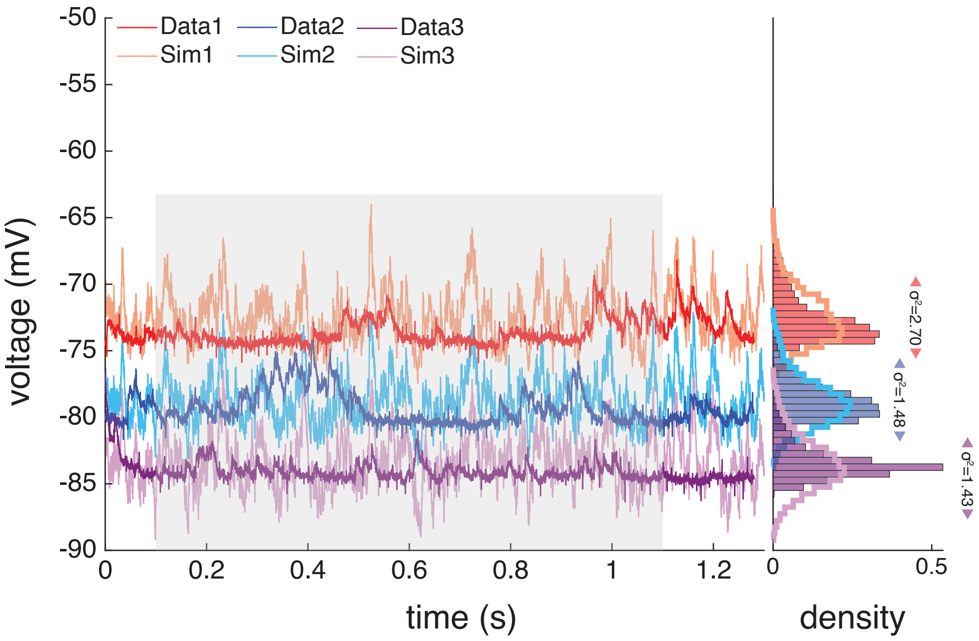

Figure R2: **Example subthreshold voltage fit.** Spontaneous subthreshold recordings from awake primates in an hyperpolarized nonspiking neuron. Input parameters $(K_{\mathrm{e}}, K_{\mathrm{i}}, \rho_{\mathrm{e}}, \rho_{\mathrm{i}}, \rho_{\mathrm{ei}})$ were estimated from assuming moderate synaptic weights and low baseline firing rates ($r_{\mathrm{e}} = r_{\mathrm{i}} = 1\mathrm{Hz}$). Simulation traces were generated using the conductance-based model with estimated parameters: $K_{\mathrm{e}} = 20,320$, $K_{\mathrm{i}} = 21,115$, $\rho_{\mathrm{e}} = 0.002$, $\rho_{\mathrm{i}} = 0.001$, and $\rho_{\mathrm{ei}} \simeq 0$. Red, blue, and purple traces correspond to hyperpolarizing input currents of $-0.06\mathrm{nA}$, $-0.14\mathrm{nA}$, and $-0.2\mathrm{nA}$, respectively, with an assumed input resistance of $200\mathrm{M}\Omega$ in all cases.

was not dependent on the occurrence of action potentials, indicating that it was not caused by mutual interconnections." That said, we do agree with the reviewer that post-spiking reseting must play a role in highly active areas.

Following one the reviewer's request, we included a paragraph in the discussion that summarizes these points.

Lastly, about connection with experimental time traces. Comparison with real data is, to my mind, a necessity on PLoS Computational Biology articles, and would add substantial value to the present article. However, as I already wrote in my comments, this is ultimately a matter of editorial policy.

We believe that the reviewer's persistent concern about comparison with real data is the result of an unfortunate misunderstanding. In the following, we explain why we believe we can rebut this concern altogether.

Our analysis is motivated by the fact that classical modeling approaches do not satisfactorily reproduce certain measurements about neuronal variability that are readily available in the existing literature. Specifically, the measurements of interest to our study are the variance and skewness of the membrane voltage in single neurons [1, 2, 3, 4, 5], as well as the crosscorrelation between membrane voltages across pairs of neurons [6, 7, 8, 9, 10]. We extensively cite experimental works reporting these measurements in awake animals throughout the manuscript and use these measurements to calibrate our models (see, e.g., Section "Biophysical parameters") and to assess the merits of our results about the role of input synchrony (see, e.g., Section "Impact of synchrony on voltage covariability"). For these reasons, we believe that our work already includes direct comparisons with experimental data in its current form, as its delivers models that can jointly account for the order of magnitude of the mean, variance, skewness and covariance of membrane potentials in response to correlated synaptic inputs with realistic, quantitatively assessed, levels of synchrony.

We are therefore a bit confused about Reviewer 3's comment about including additional "comparison with real data". In our first response, we interpreted this comment as a request to include a comparison with *newly collected* data, which motivated the following reply:

"We agree with the reviewer that a direct comparison with originally collected data is of paramount importance. That said, this work is the result of a collaboration between computational neuroscientists and probabilists that builds on already published experimental data. In that respect, we would like to mention that some of us have (hopefully) addressed the reviewer's comments in a manuscript that is currently under revision in another journal with a more experimental target audience. However, this manuscript is the result of a separate collaboration and we do not have permission to include the associated data in this submission."

The mentioned collaboration can be found on bioRxiv [11]. That said, we now realized that we misinterpreted Reviewer 3's concern. We apologize for this misunderstanding but at the same time, we respectfully rebut the assessment that we do not compare our analytical results with real data.

References

[1] Matthew A Smith and Adam Kohn. Spatial and temporal scales of neuronal correlation in primary visual cortex. *Journal of Neuroscience*, 28(48):12591–12603, 2008.

[2] Matthew A Smith and Marc A Sommer. Spatial and temporal scales of neuronal correlation in visual area v4. *Journal of Neuroscience*, 33(12):5422–5432, 2013.

[3] A. Y. Y. Tan, S. Andoni, and N. J. Priebe. A spontaneous state of weakly correlated synaptic excitation and inhibition in visual cortex. *Neuroscience*, 247:364–375, 2013.

[4] Andrew Y. Y. Tan, Yuzhi Chen, Benjamin Scholl, Eyal Seidemann, and Nicholas J. Priebe. Sensory stimulation shifts visual cortex from synchronous to asynchronous states. *Nature*, 509(7499):226–229, 2014.

[5] Oren Amsalem, Hidehiko Inagaki, Jianing Yu, Karel Svoboda, and Ran Darshan. Subthreshold neuronal activity and the dynamical regime of cerebral cortex. *Nature Communications 2024 15:1*, 15:1–17, 9 2024.

[6] Ilan Lampl, Iva Reichova, and David Ferster. Synchronous membrane potential fluctuations in neurons of the cat visual cortex. *Neuron*, 22(2):361–374, 1999.

[7] James FA Poulet and Carl CH Petersen. Internal brain state regulates membrane potential synchrony in barrel cortex of behaving mice. *Nature*, 454(7206):881–885, 2008.

[8] Michael Okun and Ilan Lampl. Instantaneous correlation of excitation and inhibition during ongoing and sensory-evoked activities. *Nature neuroscience*, 11(5):535–537, 2008.

[9] Jianing Yu and David Ferster. Membrane potential synchrony in primary visual cortex during sensory stimulation. *Neuron*, 68(6):1187–1201, 2010.

[10] Sergio Arroyo, Corbett Bennett, and Shaul Hestrin. Correlation of synaptic inputs in the visual cortex of awake, behaving mice. *Neuron*, 99(6):1289–1301, 2018.

[11] Jagruti J. Pattadkal, Ronan T O'Shea, David Hansel, Thibaud Taillefumier, Darrin Brager, and Nicholas J. Priebe. Synchrony dynamics underlie irregular neocortical spiking. *bioRxiv*, 2024.

[12] Logan A. Becker, Baowang Li, Nicholas J. Priebe, Eyal Seidemann, and Thibaud Taillefumier. Exact analysis of the subthreshold variability for conductance-based neuronal models with synchronous synaptic inputs. *Phys. Rev. X*, 14:011021, Feb 2024.

[13] Baowang Li, Brandy N Routh, Daniel Johnston, Eyal Seidemann, and Nicholas J Priebe. Voltage-gated intrinsic conductances shape the input-output relationship of cortical neurons in behaving primate v1. *Neuron*, 107(1):185–196, 2020.
